# The TLR7 agonist Imiquimod promote the immunogenicity of msenchymal stem cells

**DOI:** 10.1186/0717-6287-48-6

**Published:** 2015-01-17

**Authors:** Li Zhang, Dan Liu, Dan Pu, Yanwen Wang, Li Li, Yanqi He, Yalun Li, Lei Li, Weimin Li

**Affiliations:** Department of Respiratory Medicine, West China Hospital, Sichuan University, Cheng Du, Sichuan Province 610041 PR China; Department of Pathology, West China Hospital, Sichuan University, Cheng Du, Sichuan Province 610041 PR China

**Keywords:** Mesenchymal stem cells, Umbilical cord, Toll like receptor 7, Immunogenicity, Pro-inflammatory molecules

## Abstract

**Background:**

Mesenchymal stem cells (MSCs) are considered the best candidate in stem cells therapy due to their multipotent differentiation ability, low expression of co-stimulatory molecules (CD80, CD86, CD34 and HLA-II) and immunosuppression effects on in vivo immune responses. MSCs were now widely used in clinical trials but received no encourage results. The major problem was the fate of engrafted MSCs in vivo could not be defined. Some studies indicated that MSCs could induce immune response and result in the damage and rejection of MSCs. As toll like receptors (TLRs) are important in inducing of immune responses, in this study we study the role of TLR7 in mediating the immune status of MSCs isolated from umbilical cord.

**Results:**

Our results indicated that TLR7 agonist Imiquimod could increase the proliferation of PBMC isolated from healthy human volunteers and release of lactate dehydrogenase (LDH) in supernatant from PBMC-UCMSCs co-culture system. Flow cytometry and quantitative PCR also confirmed the regulated expression of surface co-stimulatory molecules and pro-inflammatory genes (IL-6, IL-8, IL-12, TGF-β and TNF-α). And the down-regulation expression of stem cell markers also confirmed the loss of stemness of UCMSCs. We also found that the osteo-differentiation ability of UCMSCs was enhanced in the presence of Imiquimod.

**Conclusion:**

To our knowledge, this is the first report that activation of TLR7 pathway increases the immunogenicity of UCMSCs. Extensive researches have now been conducted to study whether the change of immune status will be help in tumor rejection based on the tumor-tropism of MSCs.

## Background

Mesenchymal stem cells (MSCs) have demonstrated significant potential for clinical use. The advantages of MSCs in clinical use were due to their easily isolation, low immunogenicity, immunosuppression effects, lack of ethical controversy and potential to differentiate into tissue-specific cell types [1]. MSCs can be successfully isolated from adipose tissue, liver, tendons, synovial membrane, amniotic fluid, placenta, umbilical cord and teeth [2]. MSCs can differentiate easily into mesodermal phenotypes of adipocyte, osteoblasts, chondrocytes and some extra-mesodermal cell types including neural, pancreatic and hepatocytic phenotypes [3]. The second advantage of MSCs in cell therapy is the low expression of co-stimulatory factors including CD80, CD86, HLA-II and CD34, which enables MSCs fails to induce immune responses [4]. The third feature of MSCs is immunosuppression effects in that MSCs can inhibit the function of T cells, B cells, DC and NK cells [5]. All these advantages ensure the benefit of MSCs in clinical trials. MSCs have been used widely in regenerating damaged tissue and treat inflammation result from cardiovascular disease [6], pancreatic regeneration [7], neurological disorders [8], graft-versus-host disease (GVHD) [9] and liver fibrosis [10].

However, some recent late stage clinical trials have failed to meet the primary aims, and the fate of MSC following systemic infusion as well as the mechanism they impact on host biological functions are largely unknown [11]. Several reports indicated that engrafted MSCs could induce immune responses in vivo and cause the rejection or damage of MSCs, such as induction of memory T cell response [12], increased immunogenicity of differentiated MSCs [13], enhanced NK cell response [14] and complement response [15]. These evidences indicated that MSCs could not hold immune privilege permanently in vivo and might induce immune response under specific conditions, but large of these mechanisms remained uncovered.

Toll like receptors (TLRs) are pathogen-associated molecular patterns (PAMPs) which played important roles in mediating immune responses, especially recognize invaded pathogens by binding conserved components of microbes [16]. There are 11 family members of TLR family which recognize different pathogens [17]. Previous researches showed that TLRs were important in mediating MSCs functions. The expression of TLR1, 2, 5, 9 and 10 were significantly increased exposure to hypoxic conditions [18]. Activation of TLR4 pathway protects MSCs from oxidative stress-induced apoptosis. TLR3 agonist polyI:C increased the oseteo-differentiation [19] while TLR9 ligand CpG impaired it [20]. However, all these results of TLRs functions in MSC were from adipogenic tissues and bone marrow, no evidences were available about the role of TLRs in biological functions of MSCs isolated from umbilical cord. Although MSCs shared similarities in a lot of biological functions such as metabolic processes, biological regulation, cell communication and multicellular processes either from adipose tissues, bone marrows or from umbilical cord, there were still obvious differences between MSCs from bone marrow and umbilical cord [21]. Several embryonic stem cells (ECS) markers were present in UCMSCs, not in BMMSCs. UCMSCs expressed SSEA-1, SSEA-3, Oct-4, TRA-1-60 and DNMT3B, but showed low expression levels of genes associated with teratomas formation [22]. The UCMSCs were also found expressed higher levels of pluripotent stem cell genes (Oct-3/4, Nanog, Sox2, Sox17, Otx2 et al.) than MSCs isolated from bone marrow, adipose tissues and dental pulp [23].

As previous study indicated the activation of TLR7 increased the leukemia cells and led to the immune rejection [24]. In this study, we chose TLR7 to study whether the activation of TLR7 pathway by specific agonist Imiquimod. The aim of research is to study the role of TLR7 in regulating the immune status of UCMSCs.

## Results

### Activation of TLR7 pathway of UCMSC by Imiquimod dramatically increased the proliferation of PBMC

**Figure 1:**
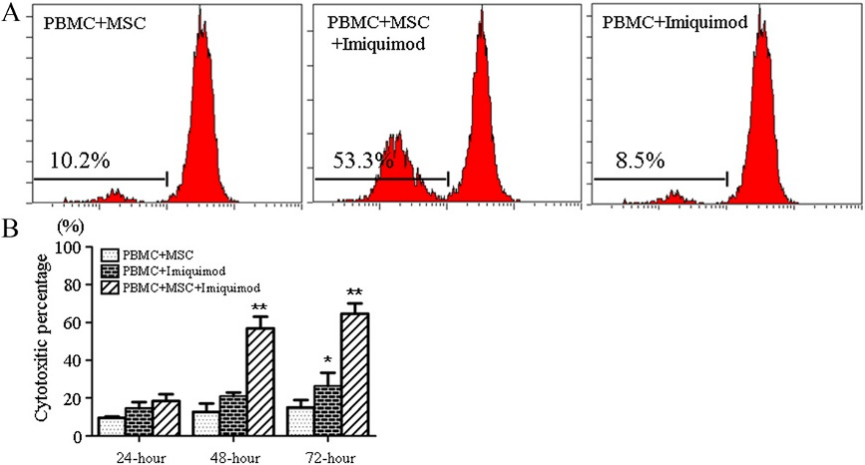
**Imiquimod increased the proliferation of PBMC and LDH release. A**: co-culture of PBMC and UCMSC, **B**: LDH detection for cytotoxicity. **P <0.001; *P <0.05 versus control group.

CFSE-labeled leukocytes proliferation was widely used to detect the increased immune responses in co-culture system. In our study, we isolated PBMC from health human volunteers and co-cultured with UCMSCs in the presence of Imiquimod, the proliferation of PBMC was 53.3% 72-hour post stimulation, while the control groups showed only 10.2% (PBMC + MSC) and 8.5% (PBMC + Imiqouid), respectively (Figure 1A). The result strongly suggested the increased immune response upon TLR7 activation.

Leukocyte-mediated cytotoxicity was a classical method to assay the LDH levels in culture medium released by damaged cells. In our experiment, we co-cultured PBMC with UCMSCs and then added Imiqouid to activate TLR7 pathway, the negative controls were PBMC co-cultured with UCMSCs without Imiquimod and the PBMC stimulated with Imiquimod. No differences were found among all three groups 24-hour post co-culture. While significantly LDH level was measured 48-hour post Imiquimod stimulation compared with control group (p < 0.001). The LDH level reached as high as 61% 72-hour post treatment in PBMC and UCMSCs co-culture group (p < 0.001), but the PBMC also showed increased LDH damage upon Imiquimod stimulation (p < 0.05) (Figure 1B). All these results confirmed the increased immunogenicity of UCMSCs activated by TLR7 agonsit Imiquimod.

### Imiquimod greatly promoted the expression of co-stimulatory molecules of UCMSCs

**Figure 2:**
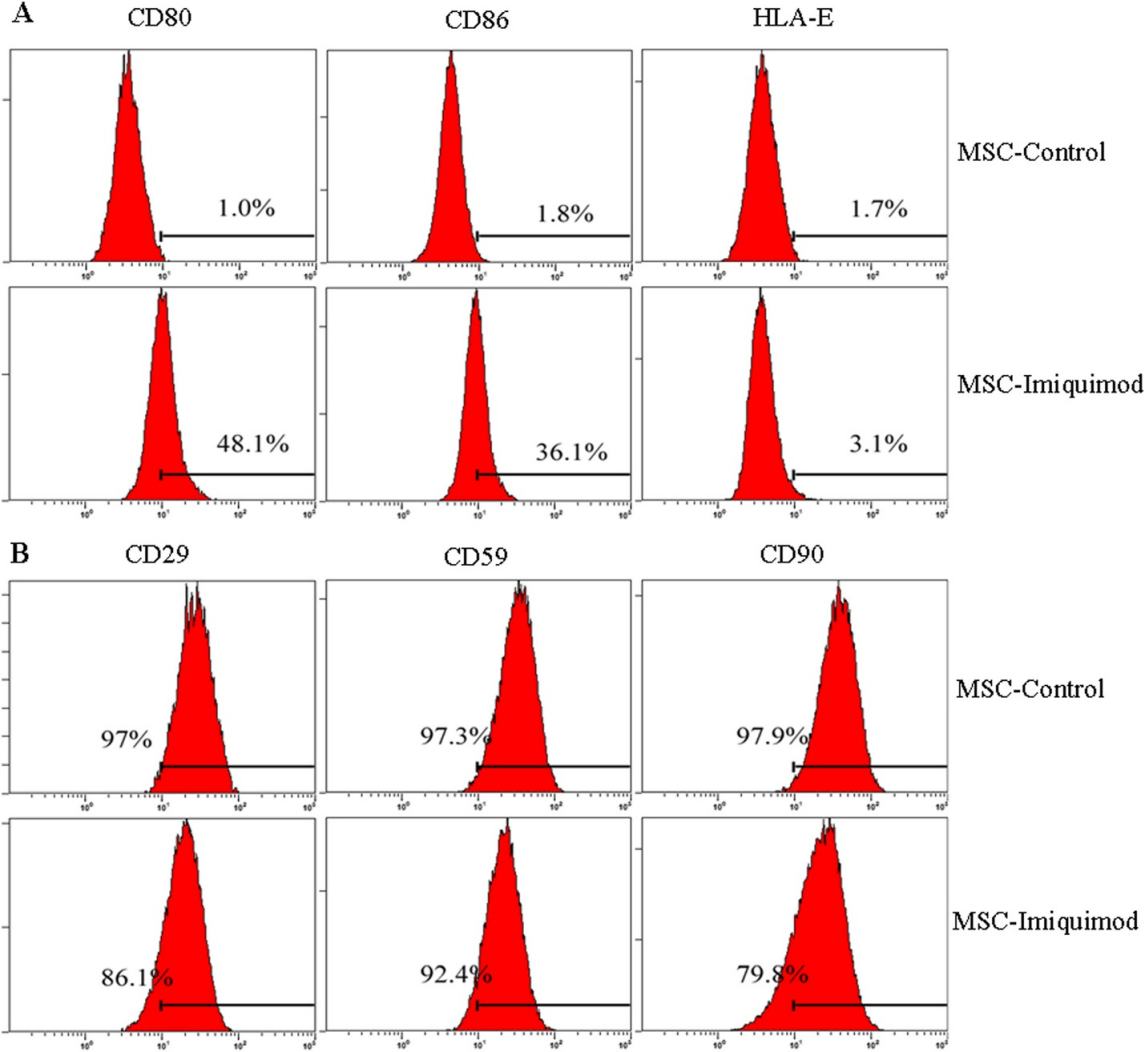
**Imiquimod enhanced the expression of co-stimulatory factors and inhibted the stem cell surface markers. A**: co-stimulatory molecules, **B**: surface markers.

CD80, CD86 and HLA-E were the co-stimulator molecules and played important role in immune responses. Here the flow cytometry detection showed that the expression of CD80 and CD86 were dramatically increased (48.1% and 36.1%) upon Imiquimod stimulation 72-hour compared with negative control (1.0% and 1.8%) (Figure 2A). While the expression of HLA-E showed almost no change (1.7% vs 3.1%) (Figure 2A). We then measured the expression of stem-cell marker CD29, CD59 and CD90 and found that expression of CD29 and CD90 were inhibited (86.1% and 79.8%) in the presence of Imiquimod (Figure 2B).

### Pro-inflammatory cytokines were induced while the stem cell markers were inhibited in the presence of TLR7 agonist Imiquimod

**Figure 3:**
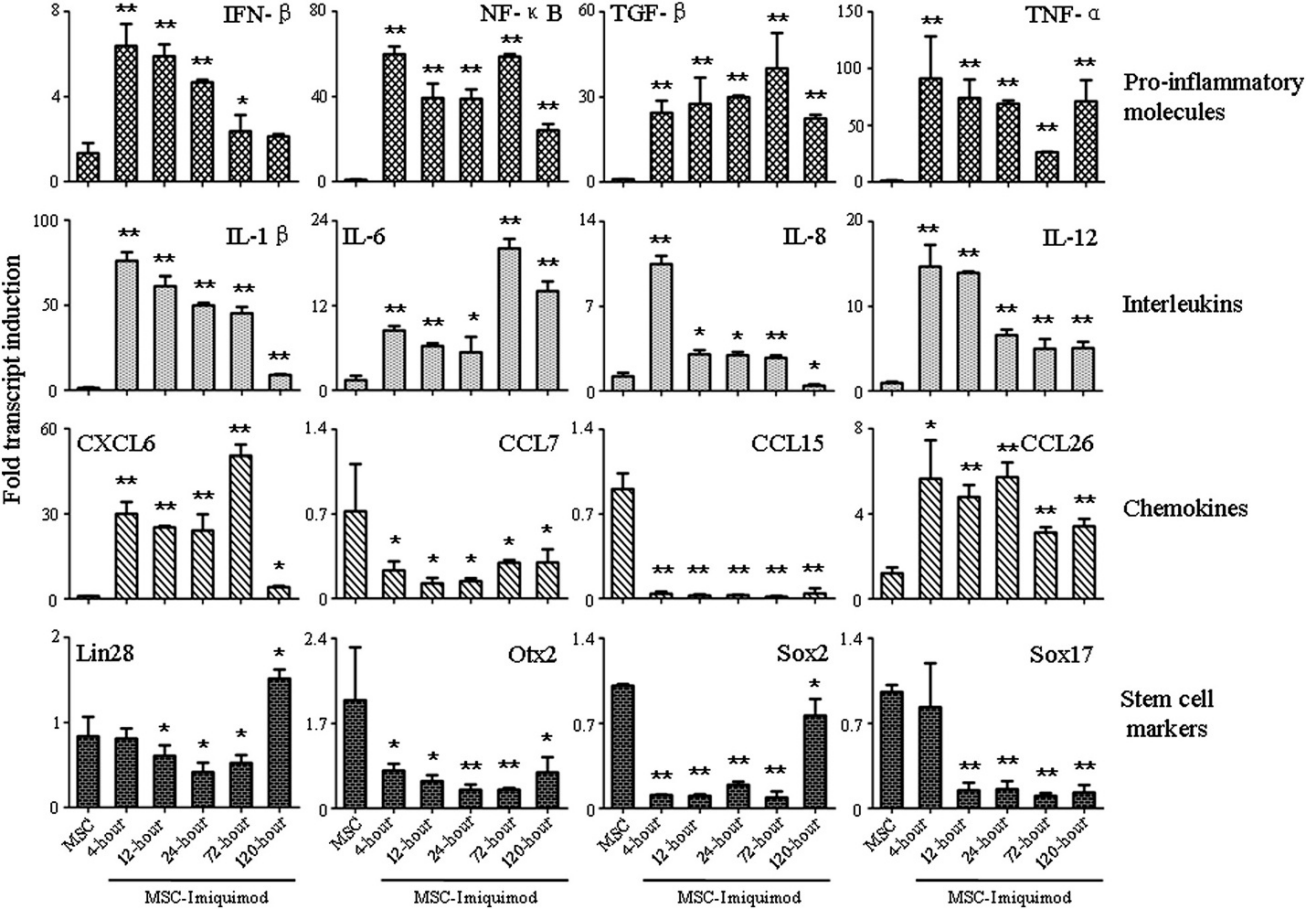
**Imiquimod induced the expression of pro-inflammatory genes but down-regulated the expression of stem cell markers.** Mean-value from three independent experiments is shown with standard derivation. **P <0.001; *P <0.05 versus control group.

The PBMC proliferation and the increased co-stimulatory molecules upon Imiquimod treatment led us to hypothesize that activation of TLR7 pathway by Imiquimod could induce the pro-inflammatory molecules which played important roles in shaping of immune status of UCMSCs. UCMSCs were treated with Imiquimod and RNA was extracted 4-hour, 12-hour, 24-hour, 72-hour and 120-hour post treatment. Quantitative PCR was conducted to measure the expression variation of important molecules in mediating the immunogenicity of cells. We found the dramatically induction of several molecules including NF-κB, TGF-β, TNF-α, IL-1β, IL-6 and IL-12 (all p < 0.001), which were know immune response-related molecules in increasing the immune responses, while in INF-β and IL-8 detection the results indicated that expressions were rapidly increased upon Imiquimod stimulation but decreased at latter time points. In chemokine detection, CXCL6 and CCL26 showed increased expression while CCL7 and CCL15 were inhibited in the presence of Imiquimod, this contradictory results indicated the complex effects of TLR7 pathway on biological functions (Figure 3).

To confirm the influence of Imiquimod on the stemness of UCMSCs, specific stem cells markers (Lin28, Otx2, Sox2, and Sox17) were tested in this study and the results indicated the significantly expression inhibition at latter time points in response to Imiquimod treatment (Figure 3). The real-time PCR of our study clearly confirmed that TLR7 agonist Imiquimod induced the pro-inflammatory phenotype of UCMSCs and reduced their stemness.

### Osteoblasts differentiation ability of UCMSCs was enhanced by Imiquimod

**Figure 4:**
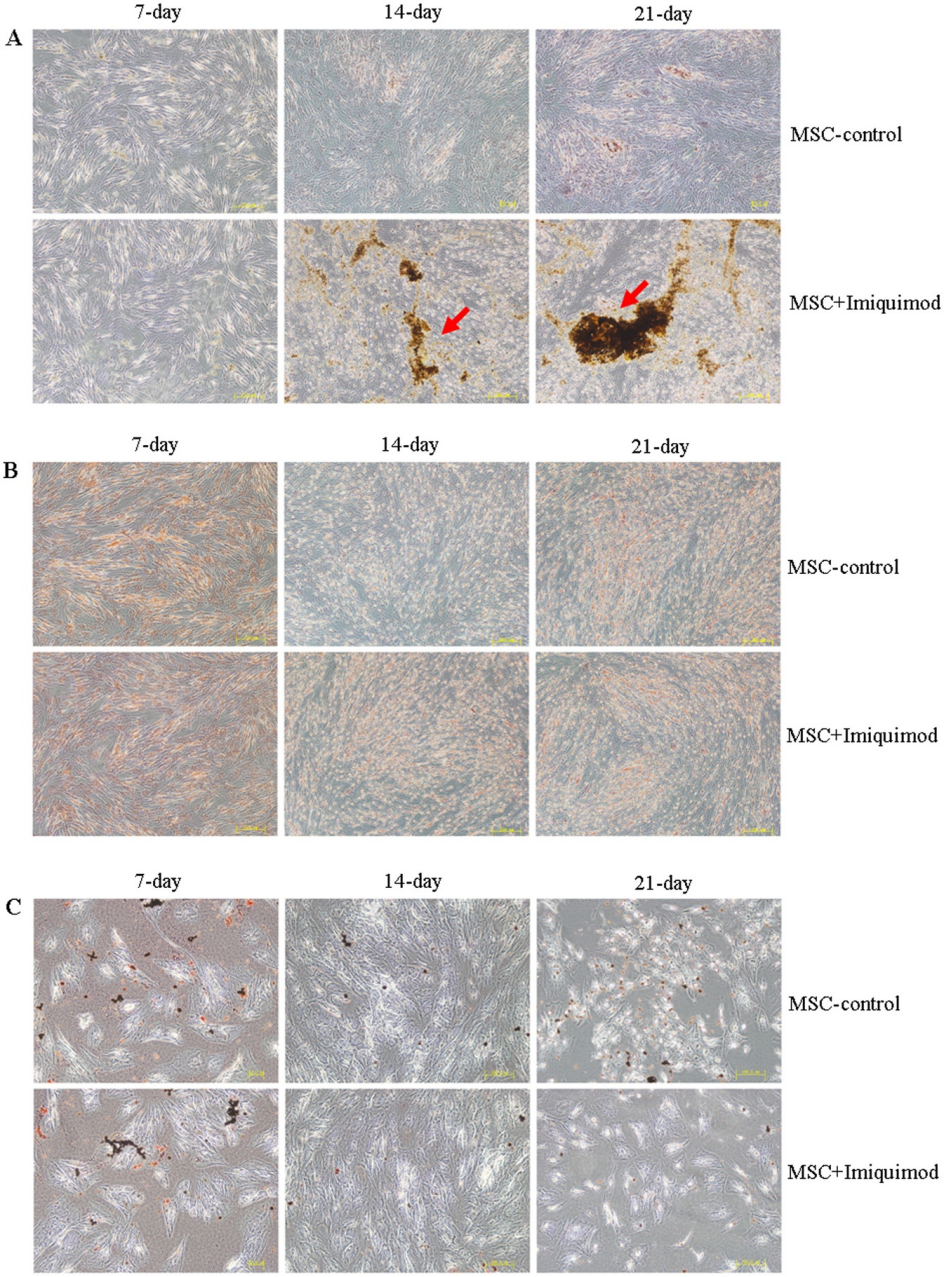
**Imiquimod enhanced the osteo-differentiation ability of UCMSCs but had no influence on adipocyte and chondrocyte differentiation. A**: Osteoblasts differentiation, **B**: chondrocyte differentiation, **C**: adipocyte differentiation.

The conditioned mediums of adipocytes, osteoblasts and chondrocytes were then added on UCMSCs in the presence of Imiquimod to test whether the activation of TLR7 pathway could influence the differentiation ability of UCMSCs. Different stain methods, as alizarin red for osteoblasts, safranine for chondrocytes, oil red O for adipocytes were performed to detect the differentiation status of UCMSCs 7-day, 14-day and 21-day post Imiquimod stimulation. We found that UCMSCs showed enhanced oste-differentiation ability in the presence of Imiquimod, as indicated by the calcium deposition (Figure 4A, red arrows). In adipocytes and chondrocytes staining, the UCMSCs showed no obvious differences between Imiquimod-treated and non-treated groups (Figure 4B and C).

## Discussion

The characteristics of MSCs including multi-potent differentiation ability, lack of expression of co-stimulatory factors and immunosuppressive effect enabled MSCs the best candidate in cell-based therapy [1]. However, previous researches indicated the induced immunogenic response in vivo against engrafted MSCs under specific conditions mediated by various molecules [11].

Toll like receptors (TLRs) played important roles in promoting immune responses. Among 11 members of TLRs, TLR7 located on the surface of endosome and could be recognized and activated by ssRNA of RNA viruses [17]. In leukemia research, activation of TLR7 by R848 could increase the immunogenicity of acute myeloid leukemia cells and result in the rejection of these cells by host immune response [24]. Previous research indicated that activation of TLRs did not change the immune status of MSCs, however, our results showed that TLR7 agonist Imiquimod promoted the immunogenicity of UCMSCs by increasing the proliferation of human PBMC in PBMC-UCMSC co-culture system. In support of this conclusion, the Imiquimod stimulation in UCMSCs increased the immune attack by human immune cells. Other evidences came from the assay of surface co-stimulatory molecules and gene expression detection, which indicated the up-regulated expression of CD80, CD86 and HLA-E on surface of UCMSCs, as well as the induced expression of pro-inflammatory cytokines (NF-κB, TGF-β, IL-6, IL-12 and TNF-α). The stem cell markers (Lin28, Otx2, Sox2 and Sox17) were also found inhibited upon Imiquimod stimulation, which confirmed the loss of stemness of UCMSCs upon TLR7 activation.

Huang et al. suggested that MSCs were immune privileged in the allogeneic heart in undifferentiated status and the differentiation of MSCs greatly increased the immune rejection by the host [13]. Based on this observation, we hypothesized that increased immunogenicity mediated by TLR7 pathway was due to the increased differentiation ability. To demonstrate our hypothesis, we put differentiation medium on UCMSCs to induce the adipocyte, osteoblast and chondrocyte from UCMSCs. The results showed that the osteo-differentiation of UCMSCs was dramatically increased by the Imiquimod stimulation. Therefore, these data confirmed our hypothesis that the increased immunogenicity of UCMSCs by TLR7 stemmed from the enhanced differentiation ability.

It is well known that MSCs can preferentially mobilize to the sites of inflammation, especially migrate to tumor tissues [25]. The mechanisms of how chemokines and cytokines are involved in MSC biological functions still remains uncovered, and awaits much more research to clarity. Previous study indicated that CCL5 and SDF-1 secreted by MSCs was responsible for enhancing the metastatic potential of several breast cancer cells [26]. And monocyte chemotactic protein-1 (MCP-1) secretion by breast cancer cells was responsible for the homing of MSCs to tumors, both locally and systemically. Irradiation of tumors released lots of inflammatory mediators, which played important roles to increase the engraftment of MSCs to the tumor [27].

## Conclusions

The activation of TLR7 pathway led to the switch of immune status of UCMSCs might play an important role in cell-based therapy, as previous report suggested that MSCs could migrate to the tumor and maintain the low immune status of tumor environment, finally protect tumor from immune responses and promote the tumor progression. So the enhanced immunogenicity of UCMSCs might cause the change of immune status in UCMSCs-tumor environment, which could be used in tumor therapy.

## Methods

### Isolation, culture and stimulation of UCMSCs

The method to isolate MSCs from umibilical cord was based on published literature [28]. The umbilical cord was diced into pieces after disinfection in 75% ethanol for 10–20 min. The small pieces of mesenchymal tissues were put into 25 mm^2^ plates. The culture medium was added when tissues sticked tightly to the bottom of plates. The dissociated MSCs were collected about 5–8 days after the mesenchymal tissues were removed. The UCMSCs were then maintained in Dulbecco’s modified Eagle’s medium (DMEM, Invitrogen, Carlsbad, CA, USA) supplemented with 10% fetal bovine serum (FBS, Invitrogen, Carlsbad, CA, USA), 2 mM glutamine, 1 mM sodium pyruvate, 100 U/ml penicillin and 100 μg/ml streptomycin and then stored in liquid for future used.

TLR7 agonist Imiquimod was purchased from IMGENEX (IMG-2207) and dissolved in DMSO at a concentration of 2.5 mg/ml. UCMSCs were seeded into 6-well plate at a concentration of 1.5 × 10^5^ in 2 ml medium and Imiquimod was added into the medium at final concentration of 10 μg/ml.

### Quantitative reverse transcript PCR

The isolation of RNA extraction, cDNA synthesis and the real-time PCR were performed according to our previous study [29]. The primers used in QPCR detection were listed in Table 1.

**Table 1:** **Primer used for real-time PCR**

Gene	Forward primer	Reverse primer	GenBank number
IL-1β	ACGAATCTCCGACCACCACT	CCATGGCCACAACAACTGAC	M15330
IL-6	GACCCAACCACAAATGCCA	GTCATGTCCTGCAGCCACTG	M14584
IL-8	CTGGCCGTGGCTCTCTTG	CCTTGGCAAAACTGCACCTT	NM_000584
IL-12	CGGTCATCTGCCGCAAA	CAAGATGAGCTATAGTAGCGGTCC	M65272
IFN-β	CAGCAATTTTCAGTGTCAGAAGCT	TCATCCTGTCCTTGAGGCAGT	M28622
NF-κB	AGAGTGCTGGAGTTCAGGATA	AAGGTGGATGATTGCTAAGTGT	AJ271718
TGF-β	TATCGACATGGAGCTGGTGAAG	CAGCTTGGACAGGATCTGGC	X02812
TNF-α	GGTGCTTGTTCCTCAGCCTC	CAGGCAGAAGAGCGTGGTG	M10988
CXCL6	GCTGAGAGTAAACCCCAAAACG	GGAGCACTGCGGGCC	NM_002993
CCL7	CTGCTCTCCAGCGCTCTCA	GTAAGAAAAGCAGCAGGCGG	NM_002984
CCL15	AGCAGAGGCTGGAGAGCTACA	GGGTCAGCACAGATCTCCTTGT	NM_006273
CCL26	CCTCTCCTGCCTCATGCTTATT	CTCTGTCTCTGCATCATTTGTGAA	U58914
Lin28	TGCTGTCGAGGGATGGATA	CCACCCAATGCGTTCTATTG	NM_024674
Otx2	TCGGGCGCAGCTAGATGT	TGTCTGGGTACCGGGTCTTG	NM_001270525
Sox2	CCCCTTTATTTTCCGTAGTTGTAT	GATTCTCGGCAGACTGATTCAA	NM_003106
Sox17	TGGCGCAGCAGAATCCA	CGACTTGCCCAGCATCTTG	NM_022454
GAPDH	GAAGGTGAAGGTCGGAGTC	GAAGATGGTGATGGGATTTC	J04038

### Leukocyte proliferation and leukocyte-mediated cytotoxicity detection

Peripheral blood mononuclear cells (PBMCs) were isolated from healthy volunteers by density centrifugation gradient and labeled by carboxyfluorescein diacetate succinimidyl ester (CFSE) at final concentration of 10 μM. Pre-cold complete DMEM was added 10 minutes to stop the reaction. CFSE-Labeled PBMCs were then washed three times by cold PBS (1200 g, 5-min). PBMCs were co-cultured with UCMSCs and collected for FACS detection 72-hours in the presence of Imiquimod.

Supernatants from PBMC-UCMSCs co-culture system were collected 24, 48 and 72-hour post stimulation and assessed by the cytotoxicity detection kit. Releasing of LDH from damaged cells was assayed according to the manual. The lysis percent was calculated by the following formula: 100 × (E-M)/(T-M), where E is experimental release, M is spontaneous release in the presence of media alone, and T is maximum release in the presence of 5% Triton X-100.

### Surface molecules detection by flow cytometry

UCMSCs, either treated or non-treated with Imiquimod, were harvested 72-hour post stimulation. The UCMSCs were then stained with different antibodies to detect surface molecules (Table 2). Flow cytometry was performed by CXP flow cytometry software. The positive standard of cells was gated according to the fluorescence intensity of negative control group.

**Table 2:** **Monoclonal antibodies for FACS analysis**

Name	Company	Catalog number
CD80	eBioscience	11-0809
CD86	eBioscience	12-0869
HLA-E	eBioscience	17-9953
CD90	eBioscience	45-0909
CD59	eBioscience	11-0596
CD29	eBioscience	17-0299

### UCMSC differentiation

UCMSCs were seeded into 6-well plate with 1.5 × 10^5^ per well. Conditioned medium of chondrocyte (GIBCO, A10071-01), osteocyte (GIBCO, A10072-01) and adipocyte (GIBCO, A10070-01) were added in each well, with 10 μg/ml Imiquimod. And oil-red was used for staining of adipocyte, alizarin red for osteocyte and safranine for chondrocyte 7-day, 14-day and 21-day.

### Data analysis

All data analysis and figures completion was the same with our previous study [29].
